# Long-term follow-up of patients undergoing laparoscopic surgery for phaeochromocytoma

**DOI:** 10.1093/bjsopen/zrac076

**Published:** 2022-06-27

**Authors:** Patrick J O’Dwyer, Cindy Chew, Samer Zino, Michael G Serpell

**Affiliations:** School of Medicine, Dentistry and Medicine, University of Glasgow, Glasgow, UK; Department of Radiology, University Hospital Hairmyres, Glasgow, UK; Department of Surgery, Ninewells Hospital, Dundee, UK; Department of Anaesthesia, University of Glasgow, Glasgow, UK

## Abstract

**Introduction:**

Phaeochromocytomas are rare tumours with a recurrence after open surgery ranging between 6–23 per cent. Long-term follow-up studies after laparoscopic surgery for phaeochromocytoma are lacking. The aim of this study was to look at the long-term oncological outcome of a consecutive series of patients from a single centre undergoing laparoscopic surgery for a phaeochromocytoma.

**Methods:**

Demographic data on all patients with an adrenal tumour or paraganglioma were prospectively kept on a database between September 1999 and December 2017. Electronic hospital records, including imaging from a national linked archiving and communication system, were reviewed for patients with a phaeochromocytoma in November 2021.

**Results:**

During the study interval 135 patients with a phaeochromocytoma were operated on in the unit, of which 118 (87.4 per cent) were attempted laparoscopically. Five (4.2 per cent) were converted to open surgery, whereas 117 had a potentially curative operation. There was no peri- or postoperative mortality. At a median follow-up of 10 (interquartile range 6–12.9) years, only 3 (2.6 per cent) patients died from metastatic phaeochromocytoma. One further patient developed lymph node metastases, which were removed at open surgery. No patient had a local recurrence and the only significant predictor of recurrence was the presence of lymph node metastases (*P* < 0.001). Two patients developed a contralateral adrenal phaeochromocytoma, while one of these also had a paraganglioma. The Kaplan–Meier estimate of phaeochromocytoma-free survival was 96 per cent (95 per cent c.i. 92.2 to 98.8) at 5 years and 92 per cent (95 per cent c.i. 86.7 to 97.3) at 10 years.

**Conclusion:**

This study demonstrates that long-term oncological outcomes of laparoscopic surgery for patients with a phaeochromocytoma are at least as good as that with an open operation.

## Teaser

Long-term follow-up studies for phaeochromocytoma are lacking. This study demonstrates that the long-term oncological outcomes for laparoscopic surgery are at least equivalent to open operation for phaeochromocytoma.

## Introduction

Phaeochromocytomas are rare tumours that arise from the chromaffin cells of the adrenal medulla or in the ganglia of the autonomic nervous system. They secrete catecholamines and are said to be the cause of hypertension in 1 per cent of patients^1^. Around 10–20 per cent of these tumours are inherited and all are treated by surgical removal where possible^2^. Recurrence after open operation has been well documented and has been thought to be between 6 and 23 per cent^3–6^. These recurrences can be malignant or benign. Benign recurrence is usually in the adrenal bed after what was thought to be a successful operation and can occur many years following the initial operation. Malignant recurrence can be in local lymph nodes or in lung, liver, or bone.

Access to both the adrenals and the ganglia of the autonomic nervous system can be difficult at open operation because of their location. Laparoscopic surgery for adrenalectomy was first described in 1992 and offers an excellent approach for both locations^7^. The views obtained at laparoscopy facilitate this approach; however, long-term follow-up studies on oncological outcomes compared with open operations are lacking.

The aim of this study was to look at the long-term oncological outcomes of consecutive patients undergoing laparoscopic surgery for phaeochromocytoma in a single centre.

## Methods

All operations for an adrenal tumour or a paraganglioma between September 1999 and the end of December 2017 were recorded on a database. To ensure completeness of data, paper, and multidisciplinary team records were also kept. For this study, only those with a catecholamine-producing tumour of the adrenal gland or a paraganglioma were included. Non-functioning paraganglioma were excluded. Data collected include date of presentation, age, sex, family history, tumour size, lymph node status, type of operation, open, or laparoscopic approach and, if laparoscopic, whether it was converted. The laparoscopic approach for both the adrenal and retroperitoneal paraganglioma was transabdominal and has been described previously^8–10^. Patients had 24-h urinary catecholamines and plasma metanephrines measured as well as CT of chest, abdomen, and pelvis and a metaiodobenzylguanidine (MIBG) scan performed before surgery. Patients were chemically blocked – initially with oral phenoxybenzamine and then intravenous phenoxybenzamine 24 h before surgery. Intraoperative hypertensive episodes were treated with intravenous phentolamine and tachycardia was managed with intravenous labetalol.

Patient follow-up was undertaken in an endocrinology clinic and data were available on the hospital electronic records system. Patients were discussed at a multidisciplinary meeting that included a pathologist and oncologist with a special interest in endocrine tumours. A phaeochromocytoma of the adrenal gland score (PASS) was recorded by the pathologists^11^. Genetic testing and counselling were undertaken for all those with a family history or bilateral phaeochromocytoma and all with a paraganglioma. Urinary catecholamines and plasma metanephrines were measured after surgery and annually thereafter. Elevated levels were investigated by an MIBG scan and CT of the chest, abdomen, and pelvis if abnormal. Whole-body MRI was conducted for patients under 20 years old. All imaging was followed on a national linked picture archiving and communication system (PACS). A consultant radiologist performed a detailed review of scans for patients with a possible recurrent phaeochromocytoma. All data were retrospectively reviewed and updated by the authors in November 2021.

### Statistical analysis

Normally distributed data are expressed as mean and s.d. Otherwise, data are expressed as median and interquartile range (i.q.r.). In frequency calculations, a chi-squared test, or chi-squared test with Yates’ correction for small numbers was applied. Phaeochromocytoma-free survival was measured by the Kaplan–Meier method. All statistics were analysed with SPSS^®^ version 20 (IBM, Armonk, New York, USA).

## Results

During the study interval 472 patients underwent 504 (32 bilateral lesions) operations for tumours of the adrenal gland or paraganglioma. Of these, 135 (28.6 per cent) were for phaeochromocytoma, of which 118 (87.4 per cent) were attempted laparoscopically. Demographic data of the 118 patients are shown in *Table 1*. Patient age ranged between 15–82 years and tumour size ranged between 5–150 mm. The reasons for open operations are shown in *Table 2*; on no occasion was this based on tumour size. All paragangliomas in the first years of the unit experience were performed as an open procedure, whereas after that all were attempted laparoscopically. Five (4.2 per cent) were converted to open operation and seven had synchronous laparoscopic operations for symptomatic gallstones (four), renal cancer (one), ovarian dermoid (one), and incisional hernia repair (one). Of the 18 patients with a paraganglioma, two had lymph node metastases at operation. One patient with an adrenal phaeochromocytoma also had lymph node metastases and eight had a PASS score of 4 or more. The difference in lymph node metastases between adrenal and paraganglioma phaeochromocytomas was significant (*P* = 0.010).

**Table 1:** Demographics of patients undergoing laparoscopic surgery for phaeochromocytoma

Patient characteristics (*n* = 118)	
**Age (years), median (i.q.r.)**	50 (37–65)
**Sex ratio (male:female)**	50:68
**Adrenal tumours**	100
**Paraganglioma**	18
**Bilateral tumours**	7
**Tumour size (mm), mean (s.d.)**	51.2 (28.6)
Familial incidence of tumours*	24
**Recurrent disease**	4
**Metastatic disease**	1
**Synchronous cancers at presentation†**	4

* VHL, Von Hippel–Lindau (*n* = 9); MEN, multiple endocrine neoplasia type IIA or IIB (*n* = 7); SDH, succinate dehydrogenase B or D gene mutation (*n* = 5); NF 1, neurofibromatosis I (*n* = 2).

†Breast cancer (*n* = 1); medullary cancer of thyroid (*n* = 1); renal cancer (*n* = 1); diffuse B cell lymphoma (*n* =1). i.q.r., interquartile range.

**Table 2:** Reasons for open operations for phaeochromocytoma

**Paraganglioma (retroperitoneal)**	6
**Urinary bladder paraganglioma**	3
Adrenal phaeochromocytoma*	8

* Synchronous large incisional hernia repair (*n* = 2); recurrent disease (*n* = 2); local invasion of vena cava (*n* = 2); ischaemic colitis (*n* = 1); palliative procedure for synchronous liver metastasis (*n* = 1).

No perioperative or postoperative mortality was observed. At a median follow-up of 10 (i.q.r. 6–12.9) years, no patient had developed a local recurrence of their phaeochromocytoma in the adrenal bed or the primary site of their paraganglioma. Three patients (2.6 per cent) died from metastatic phaeochromocytoma at 5, 5.5, and 6.2 years from their initial operation. A further patient developed lymph node metastases that were successfully removed at open operation. Two patients developed contralateral adrenal phaeochromocytomas and one of these also had a paraganglioma. The Kaplan–Meier survival estimate of phaeochromocytoma-free survival was 96 per cent (95 per cent c.i. 92.2 to 98.8) at 5 years and 92 per cent (95 per cent c.i. 86.7 to 97.3) at 10 years.

**Fig. 1:**
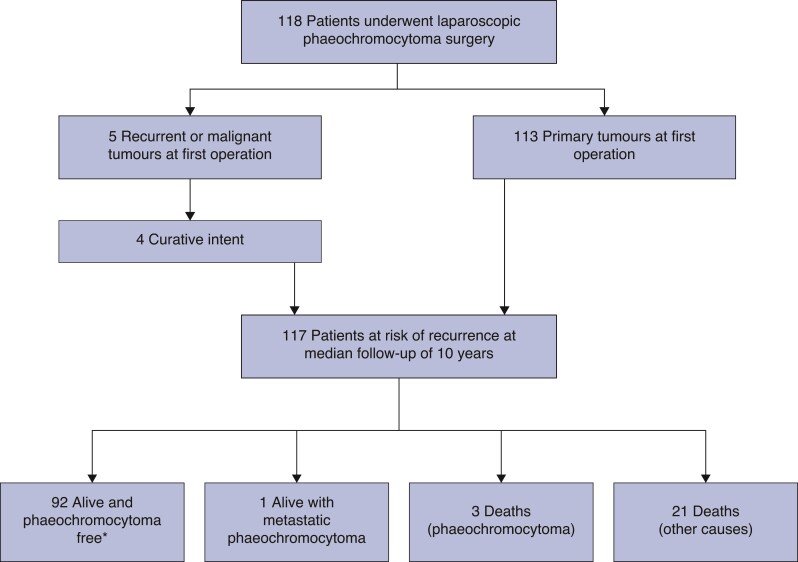
Outcome of patients undergoing laparoscopic surgery *One patient is currently receiving chemotherapy for lung cancer.

A further 21 patients died during follow-up – the causes of death included cancer (*n* = 11), cardiovascular or respiratory disease (*n* = 9), and sepsis (*n* = 1). One patient is currently alive with lung cancer and one with metastatic phaeochromocytoma (*Fig. 1*). The cancer types resulting in death are shown in *Table 3*.

**Table 3:** Cancer-related deaths during follow-up (*n* = 13)

**Phaeochromocytoma**	3
**Lung cancer**	3
**Pancreatic cancer**	1
**Melanoma**	1
**Lymphoma**	1
**Colonic cancer**	1
**Sarcoma**	1
**Oral cancer**	1
**Medullary cancer of thyroid**	1

There was no association between prognostic factors such as patient age, tumour size, PASS score, extra-adrenal location or genetic mutations, and tumour recurrence in this study; however, two of the three node-positive patients had recurrence, and this was highly significant at *P* < 0.001.

## Discussion

This study demonstrates a low mortality and recurrence rate after laparoscopic surgery for phaeochromocytoma. These findings seem better than that obtained for open operation^3–6^. In particular, there was no local recurrence after more than 1000 years of patient follow-up. As already stated, the adrenal is situated in a difficult location for open operation, particularly with a transabdominal approach. Laparoscopy on the other hand provides easy access to these sites with mobilization of the right lobe of the liver for the right adrenal and the spleen and splenic flexure of the colon for the left adrenal. This is facilitated by placing the patient on their side on the operating table, allowing these organs to fall away from the operative field. In addition, there is the added advantage of a magnified view allowing excellent assessment of the limits of the tumour. Magnification also allows easier identification of the adrenal vessels, particularly on the right side, where the vein is often short and can easily be injured. Finally the use of modern energy devices that help seal small vessels and provide an additional margin of cellular destruction is also helpful.

Another aspect of this study is the absence of intraoperative or postoperative mortality when compared with open operation. Laparoscopic surgery for phaeochromocytoma facilitates gentler handling of tissues compared with the open approach and also allows early identification of the adrenal vein so that it can be clipped with minimal mobilization. Improvements in anaesthesia and perioperative care may also be a factor.

There was no association between accepted risk factors and mortality or recurrence from phaeochromocytoma in this study^12,13^. This reflects the low number of patients with tumour recurrence in this study. It is well recognized that there is a higher recurrence rate associated with familial syndromes such as multiple endocrine neoplasia (MEN) 2A and 2B, Von Hippel–Lindau, and neurofibromatosis type 1. In addition, paragangliomas are more likely to be malignant: two of 19 patients with paraganglioma had lymph node metastases compared with one of 108 adrenal phaeochromocytomas in this study. At least 10 per cent of paragangliomas are thought to be malignant and the risk of malignancy is highest in those that carry the succinate dehydrogenase B (*SDHB*) gene mutation. Age under 20 years and tumour size of 150 mm or more are also associated with an increased risk for recurrence for phaeochromocytomas. The only significant factor in predicting recurrence in this study was the presence or absence of lymph node metastases. This has not been highlighted previously and may be related to the fact that suspicious lymph nodes were removed at laparoscopy, which may be less easily identified with an open approach.

Screening patients and families of patients with familial syndromes is now common practice^2,14,15^. Twenty per cent of patients in the laparoscopic group had familial syndromes and this is similar to that observed in a large series of patients undergoing open operation for phaeochromocytoma^6^. Some of those screened will have non-functioning tumours and this population was excluded from this study. Four individuals had functioning phaeochromocytomas identified through screening in this laparoscopic series.

While laparoscopic surgery has been shown to be safe and feasible for phaeochromocytomas, long-term follow-up studies are rare. The European Society of Endocrinology recommends that these patients should be followed for 10 years^13^. In a recent study by Zhu *et al.*, comparing laparoscopic, and open adrenalectomy for phaeochromocytoma, over a 10-year interval, they concluded that both had similar outcomes^16^; however, the interval of follow-up and how it was undertaken are not clear from the study. In a further study by Falhammar *et al*., 70 per cent of patients had laparoscopic surgery for phaeochromocytoma or paraganglioma^17^. At a median follow-up of 8 (i.q.r. 4–13) years, 13 per cent of patients died from metastatic phaeochromocytoma and they concluded that outcomes were slightly better than in previous studies. Finally, in a report by Hue *et al*. looking at the outcomes of malignant phaeochromocytoma based on an operating approach – open or laparoscopic – there was no association between operating approach and outcome on multivariate analysis^18^. As the data were extracted from the National Cancer Database (NCDB), and all phaeochromocytomas are classified as invasive in the NCDB, the findings are limited. A further limiting factor of that study is that disease-specific outcomes are not possible using NCDB data.

Some surgeons favour an endoscopic retroperitoneal approach over the laparoscopic approach. A recent meta-analysis comparing both found a shorter operating time, less blood loss, and a shorter duration of hospital stay in favour of the retroperitoneal approach for phaeochromocytomas^19^. No long-term follow-up data on local or distal recurrence with this approach are available, however.

Almost 20 per cent of patients died mainly from cancer or cardiovascular disease in this study and this may have been a factor in the low death rate from phaeochromocytomas. While life expectancy in Scotland is lower than the rest of the UK, a life expectancy of 76.79 years for men and 81.01 years for women is similar to that of other developed nations^20^.

There are several potential drawbacks of this study, one of which could be the selection process for those undergoing a laparoscopic approach. Three adrenalectomies were deemed too advanced for a laparoscopic approach; however, one of these had a large plug of tumour in the vena cava, whereas another was felt to locally invade the vena cava on imaging. The third patient had extensive liver metastases and an open approach was felt necessary to get good palliation for a patient who was under 20 years old. Other patients with adrenal lesions required a synchronous open procedure, whereas the remaining patients had paragangliomas that were treated by open operation during early experience in the unit. A second potential criticism of this study is that while all patients were managed in a single unit and baseline data were entered prospectively, follow-up was retrospective. Despite this, with electronic patient records and access to PACS, follow-up was detailed, and unlikely to have missed a key event. Furthermore, the catchment population is stable and only two patients had left the region and were possible to track through their family general practice. Finally, no attempt was made to look at non-oncological outcomes in this study, although detailed intraoperative outcomes, as well as impact on postoperative blood pressure in a smaller cohort of patients, have been published^9^.

This study demonstrates that long-term oncological outcomes of laparoscopic surgery for patients with a phaeochromocytoma are at least as good as that with the open operation. This would suggest, where possible, that patients with a phaeochromocytoma should be managed laparoscopically.

## Data Availability

Data can be made available on request to corresponding author.
